# First-Generation iStent Bypass Implantation versus ab Externo Canaloplasty Combined with Phacoemulsification in Patients with Primary Open Angle Glaucoma—12-Month Follow-Up

**DOI:** 10.3390/jcm12175711

**Published:** 2023-09-01

**Authors:** Kinga Gołaszewska, Iwona Obuchowska, Joanna Konopińska

**Affiliations:** Department of Ophthalmology, Medical University of Białystok, 15-089 Białystok, Poland; kin.golaszewska@gmail.com (K.G.); iwonaobu@wp.pl (I.O.)

**Keywords:** glaucoma, primary open angle glaucoma, iStent, canaloplasty, phacoemulsification

## Abstract

This prospective, non-randomized, interventional clinical study evaluated the efficacy and safety profile of first-generation iStent bypass implantation versus ab externo canaloplasty, both combined with phacoemulsification, in patients with primary open-angle glaucoma (POAG) after 12-month follow-up. A total of 138 patients with POAG and their 138 eyes (69 phaco-iStent and 69 phacocanaloplasty) were included. Postoperatively at 12 months, the mean intraocular pressure (IOP) decreased from 18.44 ± 3.88 to 15.51 ± 2.50 mmHg and from 17.20 ± 4.04 to 14.97 ± 2.37 mmHg in the phaco-iStent (PiS) and phacocanaloplasty (PC) groups, respectively (*p* = 0.480). In both groups, 35.7% achieved >20% IOP reduction from baseline. A total of 86% and 71.4% of the eyes were medication-free at 12 months in the PiS and PC groups, respectively. In both groups, all eyes showed improvement in best-corrected visual acuity compared with baseline and demonstrated similar safety profiles throughout the 12-month follow-up period. This study showed equal hypotensive effects of PiS and PC. Both procedures significantly reduced the IOP and the requirement for IOP-lowering medications for at least 12 months postoperatively, with no significant differences between the groups.

## 1. Introduction

Glaucoma is the leading cause of irreversible blindness worldwide. It is defined as progressive optic neuropathy characterized by damage to retinal ganglion cells and visual field (VF) defects [1]. The aim of treatment is to reduce the intraocular pressure (IOP) [2], the most important risk factor related to glaucoma onset and progression [3]. IOP depends on the balance between the rate of secretion of aqueous humor (AH) by the ciliary body and the velocity of its drainage from the eye, of which 90% occurs through the trabecular —meshwork (TM) and Schlemm’s canal (SC). Thereafter, AH enters the blood circulation. SC is a sinus venosus that connects the anterior chamber of the eye with the collector channels (CCs) and episcleral veins through the TM [4]. Several factors contribute to elevated IOP; however, in eyes with primary open-angle glaucoma (POAG), the most prevalent type of glaucoma, AH outflow, is impeded by increased resistance in various stages of the outflow pathway [5]. The maximal resistance to AH outflow is caused by the inner wall of SC (TM) [6,7].

When pharmacological treatment is insufficient in IOP control and to prevent further damage to the optic nerve, additional treatment options such as laser procedures (selective laser trabeculoplasty [SLT] and minimally invasive glaucoma surgery [MIGS]), less invasive non-penetrating glaucoma surgery (canaloplasty ab externo or deep sclerectomy), and traditional glaucoma surgeries (trabeculectomy or glaucoma drainage devices) are considered. The choice of treatment depends on the specific type and severity of glaucoma, anatomy of the affected eye, general patient health, and surgeon preference [8,9].

Trabeculectomy is the gold standard for glaucoma surgery globally and can achieve a reduction of 47–65% in IOP from baseline [10]. This bleb-dependent procedure has a high success rate but is associated with severe and frequent intraoperative and postoperative complications and long recovery after surgery. Trabeculectomy is often reserved for patients with advanced disease. The implementation of less invasive or minimally invasive antiglaucoma procedures has altered the perspective of glaucoma surgery and is increasing in cases of mild-to-moderate glaucoma [11,12]. The need for minimally invasive surgical intervention in the early stages of glaucomatous neuropathy has prompted research to seek surgeries that will improve patients’ quality of life through effective IOP control with minimal postoperative complications. A decade ago, Ahmed and Saheb proposed the term MIGS, which has gained wide interest [13]. These procedures seek to reduce IOP by targeting the source of AH outflow resistance (TM or SC) or establishing new pathways for AH outflow (to the suprachoroidal or subconjunctival spaces). Among SC-based procedures, the iStent trabecular microbypass stent (Glaukos, San Clemente, CA, USA) is widely used during phacoemulsification in patients with concomitant mild to moderate glaucoma [14,15,16,17,18,19,20,21,22]. It is designed to restore physiological AH outflow through the TM into SC. It is implanted using an ab interno approach with a clear corneal incision. The snorkel of the iStent is positioned in the anterior chamber, and the open half-pipe lumen (foot) is placed in SC to establish a patent bypass via the TM to SC and to restore continuous physiological outflow. Several studies have confirmed its effectiveness and safety profile [14,15,16,17,18].

Canaloplasty ab externo is a dynamic field in glaucoma surgery, introduced in 2007 [23]. Since then, several canaloplasty procedures have been developed, and many modifications to the original technique have been proposed. It is considered safe and effective [24,25,26,27]. Canaloplasty ab externo also affects the physiological mechanisms of AH drainage. It widens the lumen of SC and enhances the tension of its walls. It improves the physiological outflow systems at three main resistance sites, specifically, the pathology of the TM, collapse of the SC, and closure of the CCs [25]. An additional mechanism of the IOP-lowering effect is the drainage of the AH through the trabeculo-Descemet’s membrane (TDM) to the intrascleral lake, which allows prolonged absorption into the intrascleral and suprachoroidal spaces in a mechanism analogous to that observed with deep sclerectomy. This is achieved through the formation of a decompression space under the superficial scleral flap, which serves as a reservoir for AH, and thus, allows extended AH absorption from this space. This is possibly due to increased AH percolation through the reduced scleral thickness after deep flap excision under the superficial flap. However, the exact role of the intrascleral lakes remains unclear [28]. Moreover, as this surgery requires dissection of two scleral flaps and insertion of a microcatheter into SC, it is not considered as MIGS.

The present study can contribute to our understanding of resistance to physiological outflow mechanisms in glaucoma pathology by comparing the efficacy and safety of two SC-based procedures: iStent implantation and canaloplasty ab externo, both combined with phacoemulsification. Several studies have found both methods to be effective and safe in glaucoma therapy. However, to our knowledge, this is the first study to compare the clinical setup of these two types of glaucoma procedures with 12 months of follow-up.

## 2. Materials and Methods

### 2.1. Protocol

This prospective, single-center, non-randomized, interventional clinical study was approved by the Bioethics Committee of Bialystok Medical University (consent No.: APK.002.278.2021) before study initiation. Participants were enrolled between February 2021 and May 2022; they provided informed consent to participate in the study and a declaration of attendance at scheduled control visits within 12 months of operation. This trial included consecutive patients with glaucoma assigned to undergo first-generation phaco-iStent (PiS) bypass implantation or ab externo phacocanaloplasty (PC), both combined with phacoemulsification. This study was conducted at the Department of Ophthalmology at the Medical University of Bialystok, Poland, in accordance with the principles of the Declaration of Helsinki. A single surgeon performed all the procedures (J.K.).

The criteria for study eligibility, visits, and procedures were implemented in line with those of recent trials evaluating the outcomes of antiglaucoma procedures [29,30] and have been previously outlined [31]. In summary, the study included participants aged ≥21 years with visually significant cataracts and mild-to-moderate POAG, defined according to the ICD-10 guidelines. The participants had a mean VF deviation of −12 dB or better, as determined by the results of the preoperative 30-2° Humphrey VF test (Carl Zeiss Meditec Dublin, CA, USA). The severity of glaucoma was determined based on Hodapp’s classification according to the European Glaucoma Society (EGS) [8] guidelines as follows: early (MD > −6 dB), moderate (MD −6 to −12 dB), or advanced (MD < −12 dB). Surgery was indicated for patients who could not achieve the target IOP despite receiving the maximum tolerated pharmacological treatment and, in whom glaucoma progression was detected on three standard automated perimetry (SAP) examinations within a year. Newly diagnosed patients underwent SAP examinations thrice per year during the first two years after diagnosis to assess the progression of glaucomatous neuropathy. Exclusion criteria included anterior chamber angle less than a Schaffer grade 3 in all four quadrants as determined by gonioscopy, presence of advanced glaucoma, any type of glaucoma other than POAG, had SLT within 3 months prior to enrollment, history of any glaucoma surgery, unwillingness to participate in the study, and one or both eyes with best-corrected visual acuity (BCVA) less than a “finger count” [31].

The primary outcome measures were reduction in IOP and medication burden. The secondary outcome measures were BCVA, mean VF deviation, endothelial cell density (ECD), and adverse events. IOP was assessed using Goldmann tonometry by an experienced operator. At each measurement time point, two readings were taken, and their averages were recorded. If the difference between the two readings exceeded 2 mmHg, a third reading was obtained, and the median value was recorded. Participants who met all the eligibility criteria underwent surgery within 14 days.

We defined the outcome of complete surgical success when no additional glaucoma surgeries were required, IOP was ≤15 mmHg, and all antiglaucoma medications were discontinued. Qualified surgical success was characterized by an absence of the need for further glaucoma surgeries and an IOP ≤ 15 mmHg, regardless of whether antiglaucoma medications were discontinued. We categorized surgical failure as the requirement for another glaucoma surgery following the initial procedure or an IOP > 15 mmHg during two consecutive control visits despite the administration of antiglaucoma medications.

The choice of procedure, either iStent implantation or ab externo canaloplasty, was multifactorial and influenced by both clinical and patient-specific considerations. The surgeon’s experience and anatomical characteristics of the eye play central roles in this decision-making process. Additionally, the systemic health, age, life expectancy, and ability to tolerate the procedure under local anesthesia of the patient were considered in the surgical plan. The use of anticoagulant medications favored PiS implantation under topical anesthesia. Patients’ preferences were also considered.

### 2.2. Surgical Technique

The eyes that underwent standard phacoemulsification without complications and intraocular lens (IOL) implantation in the capsular bag were subjected to 360° canaloplasty or iStent implantation. The eyes with intraoperative complications related to the cataract/IOL implantation were excluded from the glaucoma procedure. This surgical technique has been previously described [31,32,33]. Briefly, PC was performed under local retrobulbar anesthesia using 3.5 mL of 2% xylocaine solution. A corneal tensioning suture was placed in the 12 o’clock position to rotate the eyes downward. The conjunctiva and Tenon’s capsule were incised in the limbus to expose the sclera. A superficial flap of approximately 5.0 × 5.0 mm^2^ was cut to about one-third of the scleral thickness. A smaller, deep scleral flap (approximately 4.0 × 4.0 mm^2^) was dissected just above the choroidal tissue. The flap was extended forward into the clear cornea by approximately 2 mm, creating a TDM. The microcatheter was inserted into the SC and carefully guided through the canal until the tip appeared at the other end. A 10-0 polypropylene suture (Prolene; Ethicon, Inc.) was attached to the microcatheter tip to form a double loop. Viscocanalostomy with the additional passage of a microcatheter (iTrack 250A, iScience Interventional, Menlo, Park, CA, USA) through the SC was performed (360°) with a viscoelastic injection every 2 h to dilate the canal. The suture was placed under tension, and locking knots were added. The superficial flap was sutured in a watertight manner. The conjunctiva was sutured over the limbus.

The iStent implantation was performed using an ab interno approach without disrupting the conjunctiva or sclera. After performing phacoemulsification under local anesthesia (supplied as Alcaine drops), carbachol was introduced into the anterior chamber to constrict the pupil and enhance the visibility of the angle structures. A large amount of viscoelastic material was introduced into the anterior chamber. Both the patient’s head and the operating microscope were turned 30–45° away from the surgeon. A Swan–Jacobs gonioprism was placed in the cornea. The implant was then inserted into the TM in the inferior nasal quadrant (an area typically with the highest concentration of CCs). The appearance of a small amount of blood confirmed that the implant was positioned within SC. Subsequently, aspiration and irrigation were performed to remove viscoelastic materials and blood from the anterior chamber. Finally, the corneal incision was closed.

### 2.3. Postoperative Visits and Treatment

Patients were evaluated before surgery and on days 1, 7, and 14, and 1, 3, 6, and 12 months postoperatively. Postoperative care for all patients included topical antibiotic for two weeks, topical nonsteroidal anti-inflammatory drugs, and a gradually tapered topical steroid regimen for four weeks. Antiglaucoma eye drops were discontinued on the day of surgery. If the target IOP was not reached postoperatively, antiglaucoma medication was resumed according to the EGS guidelines [8].

### 2.4. Statistical Analysis and Study Endpoints

Statistical analysis was performed using R statistical software, version 4.1.2. Categorical variables are summarized as absolute numbers and % frequencies. Numerical variables are described as means and standard deviations, as well as medians and interquartile ranges. Groups were compared using Student’s independent t-test, Mann–Whitney U test, Pearson Chi-square test, or Fisher’s exact test, as appropriate. Normality was assessed using the Shapiro–Wilk test combined with skewness and kurtosis indicators. Variance homogeneity was verified using Levene’s test. All statistical tests assumed significance level of α = 0.05.

The primary efficacy outcome of this study was the decrease in IOP from baseline to 12 months postoperatively. Two recent pivotal MIGS trials involving canaloplasty and PiS procedures reported an approximate reduction of 3.5 mmHg in IOP over 12 months. Hence, a mean decrease of 3.5 mmHg in IOP at 12 months was selected as the minimum reduction expected. A sample size of 58 eyes was needed to achieve 90% power to detect a reduction of >3.5 mmHg in unmedicated diurnal IOP, based on the results of a paired t-test with a two-sided significance level of 0.05. The second primary efficacy outcome was the decrease in the number of IOP-lowering medications from screening to 12 months postoperatively. In the control arms of the two pivotal MIGS trials, an approximate reduction of 1.0 medication was evident at 12 months. To detect a mean reduction of >1.0 medication with 90% power, a sample size of 69 eyes was required based on the results of a paired t-test with a two-sided significance level of 0.05. The first secondary efficacy outcome was the percentage of eyes with a >20% reduction in unmedicated IOP from baseline to 12 months. A sample size of 66 eyes would provide a 95% confidence level for detecting the proportion of eyes achieving an outcome greater than 73%, based on a binomial distribution.

## 3. Results

### 3.1. Characteristics and Comparison of Groups–Demographics and Selected Baseline Parameters

Patients in the PiS and PC groups did not differ in age, sex, or cataract type (*p* > 0.05). Except for the number of antiglaucoma drugs, other baseline parameters did not differ between the study groups (*p* > 0.05) (Table 1).

### 3.2. Comparison of Selected Parameters between Study Groups at Each Measurement Time Point

**Figure 1:**
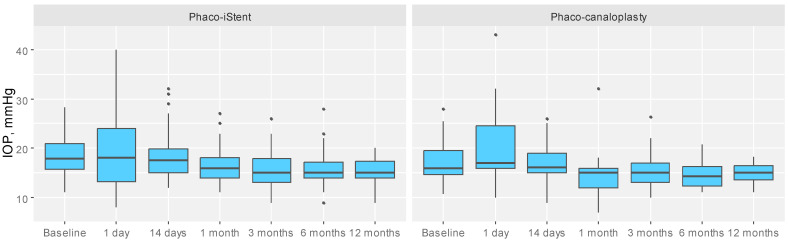
Comparison of IOP between the study groups for each measurement time point. IOP, intraocular pressure.

IOP was significantly greater in the PiS group, MD = 1.00, 95% CI [0.10;3.70], *p* = 0.020, than in the PC group after 1 month, while it did not vary between groups at baseline or any other follow-up visit (*p* > 0.05) (Table 2) (Figure 1). The proportion of patients with 20% reduction in IOP from baseline to 12 months did not differ between the groups (*p* > 0.05) (Table 3).

**Figure 2:**
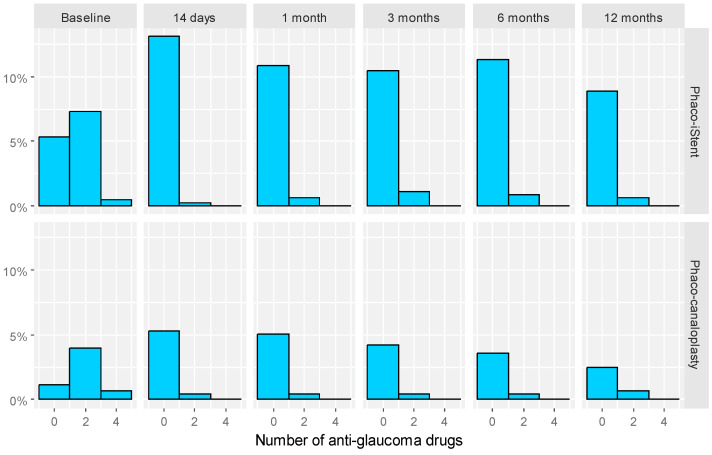
Distribution of number of antiglaucoma drugs for each measurement time point in both study groups.

There were no significant changes in the MD in VF in either of the groups during the entire follow-up period (*p* > 0.05). All eyes in both groups maintained or exhibited improved BCVA relative to the baseline (*p* > 0.05). Moreover, there were no significant differences in ECD between the two groups during the entire follow-up period. After 12 months of observation, the ECD was 1556.90 ± 614.84 and 1645.29 ± 534.56 cells/mm^3^ in the PiS and PC groups, respectively (*p* > 0.05). The number of antiglaucoma drugs varied between the groups at baseline. Patients in the PiS group were taking less antiglaucoma drugs than in the PC group, MD = −0.48, 95% CI [−0.93; −0.03], *p* = 0.036. Statistically significant differences between the groups were not identified at any follow-up visit (*p* > 0.05). Details of the number of antiglaucoma medications in the PiS and PC groups are presented in Table 4. Figure 2 presents the distribution of the number of antiglaucoma drugs for each measurement time point in both study groups.

Complete and qualified surgical success and failure were assessed at 6 and 12 months, respectively. No significant difference was found between the PiS and PC groups (*p* > 0.05) (Table 5).

Among the intraoperative and early postoperative complications (defined as complications occurring within 14 days), those that differed between the groups were microhyphema and elevated IOP, which were significantly less common in the PiS group (2.9%, *n* = 2 vs. 42.3% in the PC group, *p* < 0.001; 21.7% vs. 50.0% in the PC group, *p* = 0.015). The incidence of late postoperative complications was not significantly different between the groups (*p* > 0.05) (Table 6).

## 4. Discussion

The final outcomes of our study confirm the continued validity of the previously reported 6-month results [31] showing that both PiS and PC provide a lasting IOP-lowering effect, which resulted in significant reductions in both IOP and the necessity for IOP-lowering medications for a minimum of 12 months following surgery; the occurrence of this finding did not differ between the groups. The mean diurnal IOP was reduced by 2.93 mmHg and 2.23 mmHg in PiS and PC groups, respectively. In both groups, 35.7% patients achieved >20% IOP reduction from baseline. The average use of medication decreased by 88.89% and 71.43%, and 86% and 71.4% were medication-free at 12 months in the PiS and PC groups, respectively. No new safety issues were identified since the report of an interim analysis.

Over the years, the role of TM in glaucoma pathology has been researched extensively [5]. From a physiological perspective, the TM, especially the inner wall of SC and the TM close to the CCs, is the primary reason for resistance to AH outflow, with the external wall and surrounding tissues causing residual resistance [34,35]. This region is known as the juxtacanalicular space and is considered the main site of IOP regulation [36,37]. Zhou et al. [38] investigated the mechanical characteristics influencing resistance to AH outflow. They found that SC was highly contractile and responded to various pharmacological treatments. Drugs known to increase outflow resistance caused stiffening of SC cells, whereas drugs known to decrease outflow resistance caused softening. However, the responses to these interventions differ among patients. Interestingly, histological studies of human eyes have shown that 25–30 CCs are distributed randomly around the limbus, with a preference for the inferior nasal quadrant [39]. From these CCs, the AH flows into the aqueous veins (AVs) and through a system of venous plexuses, deep scleral plexus, limbal plexus, and intrascleral plexus, which eventually leads to the episcleral vein system [36], ocular veins, and ultimately into the general circulation [40]. AVs have lumens directly connected to CCs and episcleral veins that drain blood into the general circulation, bypassing the deep scleral and intrascleral venous plexuses [41]. AVs initially carry a clear AH and then merge with blood-filled episcleral veins, creating visible transition zones on the conjunctival surface characterized by large vessels with a transparent central lumen surrounded by dark blood [42].

Canaloplasty and iStent implantation have a similar IOP-lowering effect on the natural AH outflow pathway. The stent was designed with a pointed tip to aid insertion into SC, effectively bypassing the site of greatest resistance to AH outflow from the eye, the TM, which is associated with IOP regulation. Huang et al. reported that trabecular-targeted stents increase AH outflow in the enucleated human eye [43]. Canaloplasty acts by various IOP-lowering mechanisms. First, reducing the resistance to outflow by inserting the suture into SC increases the permeability of the TM, SC, and juxtacanalicular tissues. Another possible mechanism is the drainage of the AH through TDM into the intrascleral decompression space created under the superficial scleral flap, which serves as a reservoir and facilitates the prolonged absorption of the AH [44]. From the intrascleral lake, the AH drains into the sclera, episclera, choroid, and/or subconjunctiva in a mechanism similar to that after deep non-penetrating deep sclerectomy. Although canaloplasty has various mechanisms for IOP reduction, the magnitude of the change is comparable to that observed after iStent implantation, which functions only by bypassing the TM. This highlights the role of the distal outflow pathway and transscleral lake, which may not play a critical role in the mechanisms that underpin the pressure-lowering effect of canaloplasty. A recent study [45] reported similar conclusions when comparing different versions of canaloplasty. Remarkably, no differences were found in the IOP-reducing effect between canaloplasty ab externo with the creation of an intrascleral lake and its variants without a decompression space (canaloplasty ab interno and minicanaloplasty). An interesting aspect that emerged from the analysis of different studies is that PiS demonstrates a variable IOP-lowering effect [21,46,47,48]. The average decrease in IOP is reported from 22.9% to 32.9% [22,46,48], with the longest follow-up of 7 years [49]. Several theories have been proposed to account for this variability, including improper surgical placement within the TM, linked to the surgical learning curve [50,51]. Another theory is that there exists unknown mechanisms in the distal AH outflow pathway. Some authors suggest that these could be associated with segmental AH outflow, and therefore, the surgeon must place the PiS in the most proficient location in the TM [50]. CCs are not located at equal distances from SC among all patients. The success or failure of the procedure may be influenced by the location of the bypass in the eye (superior, inferior, nasal, or temporal quadrant). PiS surgery is performed primarily in the nasal quadrant of the eye using a clear temporal corneal approach. However, it is not clear if it is better to implant the stent in the region with the highest numbers of CCs or the region with poor outflow, because regions with poor AH outflow could be targeted by improved outflow through the SC and opening the ostia of the CC. This could lead to the assumption that greater IOP reduction can be achieved by performing trabecular MIGS in areas of initially low AH outflow [43,50].

Cataract removal plays an important role in decreasing IOP postoperatively. The average decrease in IOP after phacoemulsification is 1.4 to 3.1 mmHg in POAG [52,53,54]. Although the mechanisms of IOP decrease in angle-closure glaucoma and PXG can be easily explained [55], various theories explaining this effect have been proposed in POAG. After lens removal, the tension changes, similar to parasympathomimetic treatment [56]. Other possible mechanisms include: (1) decrease in AH production due to vitreous pull by ciliary body processes, caused by contraction of the lens capsule after phacoemulsifuccication; (2) improvement in AH outflow through the TM and SC; and (3) improvement in uveoscleral outflow, caused by increased release of prostaglandins (PG) during the procedure: PGE-1 increases the outflow of AH by the conventional route, while PGF-2 increases the outflow by the alternative route [57,58]. In our study, all patients underwent combined glaucoma surgery; therefore, this factor played an equal role in both groups.

This study has some limitations. The patients in our study were selected for glaucoma surgery without a washout period before the procedure; therefore, their baseline IOP was at maximum tolerated therapy. If these patients had undergone cataract surgery alone, we may not have attained the target IOP without the use of antiglaucoma medications. No randomization was performed prior to surgery. Other important limitations of this study include the short follow-up period, lack of clear criteria for choosing the intervention, use of first-generation PiS, and an additional IOP-lowering effect of phacoemulsification in both groups, which might act as a confounder. In contrast, our study groups were very homogeneous, with mild-to-moderate POAG, which eliminated variations in the glaucoma course and reaction to the procedure. Therefore, despite these limitations, we believe that this study provides evidence for the safety and efficacy of both surgeries.

## 5. Conclusions

Canaloplasty and PiS procedures combined with phacoemulsification allow safe and stable IOP control. These results emphasize the importance of the TM in AH outflow resistance. By highlighting these cutting-edge surgical alternatives, we hope to contribute to the evolving landscape of glaucoma treatment and offer new hope to patients in the fight against this sight-threatening disease.

## Figures and Tables

**Table 1:** Demographics and baseline characteristics of the study groups.

Variable	Phaco-iStent		Phacocanaloplasty		MD (95% CI)	*p*
	*n* (%)/Mean ± SD	Median (Q1; Q3)	*n* (%)/Mean ± SD	Median (Q1; Q3)		
*n*	69 (100.0)	-	69 (100.0)	-	-	-
Age, years	71.49 ± 9.43	72.00 (68.00; 78.00)	70.20 ± 6.93	71.00 (64.00; 77.00)	1.29 (−2.83; 5.42)	0.535
Sex, female	51 (73.9)	-	50 (73.1)	-	-	>0.999 ^2^
Cataract						
NC1	30 (44.1)	-	37 (53.8)	-	-	0.314 ^2^
NC2	21 (30.9)	-	11 (15.4)	-		
NC3	17 (25.0)	-	21 (30.8)	-		
ECD, cells/mm^2^	2280.88 ± 408.65	2313.00 (2053.50; 2524.50)	2337.35 ± 361.90	2482.00 (2092.75; 2618.50)	−56.46 (−241.47; 128.54)	0.545
Number of antiglaucoma drugs	1.86 ± 0.94	2.00 (1.00; 3.00)	2.35 ± 1.02	2.00 (2.00; 3.00)	−0.48 (−0.93; −0.03)	0.036
BCVA, Snellen 5 m	0.63 ± 0.29	0.60 (0.40; 1.00)	0.65 ± 0.34	0.70 (0.50; 0.98)	−0.10 (−0.20; 0.10)	0.758 ^1^
IOP, mmHg	18.44 ± 3.88	17.85 (15.70; 20.92)	17.20 ± 4.04	15.85 (14.72; 19.55)	2.00 (−0.30; 3.00)	0.098 ^1^
CCT, microns	537.23 ± 43.91	529.00 (502.00; 554.75)	524.40 ± 23.66	524.00 (504.00; 536.00)	12.83 (−7.13; 32.80)	0.203
VF MD, dB	−6.69 ± 7.23	−4.66 (−6.65; −2.88)	−9.71 ± 7.33	−8.25 (−14.93; −3.83)	3.59 (−1.49; 9.43)	0.267 ^1^

Notes: SD, standard deviation; Q1, 1st quartile; Q3, 3rd quartile; MD, mean or median difference; CI, confidence interval; NC; ECD, endothelial cell density; BCVA, best-corrected visual acuity; IOP, intraocular pressure; CCT; VF, visual field. Data presented as *n* (%) for nominal variables and mean ± SD with median (Q1; Q3) for numerical variables. Groups were compared using Student’s independent *t*-test, Mann–Whitney U test ^1^, and Pearson chi-square test ^2^.

**Table 2:** Comparison of IOP between study groups.

IOP, mmHg	Phaco-iStent	Phacocanaloplasty	MD (95% CI)	*p*
Baseline	18.44 ± 3.88	17.20 ± 4.04	2.00 (−0.30; 3.00)	0.098 ^1^
1 day	19.79 ± 7.90	20.03 ± 7.43	1.00 (−4.10; 5.00)	0.872 ^1^
14 days	18.11 ± 4.57	17.02 ± 4.06	1.35 (−1.00; 2.70)	0.476 ^1^
1 month	16.58 ± 3.60	14.64 ± 4.57	1.00 (0.10; 3.70)	0.020 ^1^
3 months	15.51 ± 3.27	15.40 ± 3.77	0.00 (−1.10; 2.00)	0.737 ^1^
6 months	15.87 ± 3.34	14.62 ± 2.77	0.75 (−0.50; 2.90)	0.192 ^1^
12 months	15.51 ± 2.50	14.97 ± 2.37	0.54 (−0.98; 2.06)	0.480

Notes: IOP, intraocular pressure; MD, mean or median difference; CI, confidence interval. Data are presented as mean ± SD. Groups were compared using the Student’s independent *t*-test or Mann-Whitney U test ^1^.

**Table 3:** Comparison of proportion of patients with >20% reduction in IOP from baseline to 12 months between the phaco-iStent and phacocanaloplasty groups.

Variable	Phaco-iStent	Phacocanaloplasty	*p*
>20% reduction in IOP from baseline to 12 months	15 (35.7)	15 (35.7)	>0.999

IOP, intraocular pressure. Notes: Data are presented as *n* (%). Groups were compared using Pearson’s chi-square test.

**Table 4:** Number of anti-glaucoma medications in the phaco-iStent vs. phacocanaloplasty groups.

	Phaco-iStent		Phacocanaloplasty	
Variable	1 Month	12 Months	1 Month	12 Months
Number of antiglaucoma medications, mean ± SD	0.12 ± 0.47	0.21 ± 0.56	0.16 ± 0.55	0.64 ± 1.15
Reduction (vs. baseline), mean ± SD	1.71 ± 0.99	1.51 ± 1.07	2.16 ± 0.99	1.71 ± 1.33
Reduction (vs. baseline), %, mean	95.35	88.89	95.14	71.43
Medication discontinuation, *n* (%)	49 (94.2)	37 (86.0)	63 (92.0)	49 (71.4)
No increase in IOP (vs. baseline) out of eyes without medication, *n* (%)	30 (62.5)	26 (72.2)	45 (65.2)	48 (70.0)
No increase in medications (vs. baseline), *n* (%)	45 (100.0)	34 (97.1)	69 (100.0)	59 (85.7)

Notes: SD, standard deviation; IOP, intracular pressure.

**Table 5:** Comparison of surgical success/failure between phaco-iStent and phacocanaloplasty groups.

Variable	Phaco-iStent	Phacocanaloplasty	*p*
Complete surgical success			
After 6 months	41 (75.9)	57 (82.4)	0.745 ^1^
After 12 months	33 (76.7)	48 (69.2)	0.717 ^1^
Qualified surgical success			
After 6 months	44 (81.5)	65 (94.1)	0.277 ^1^
After 12 months	38 (88.4)	58 (84.6)	0.658 ^1^
Surgical failure			
After 12 months	5 (11.6)	5 (7.7)	>0.999 ^1^

Notes: IOP, intraocular pressure. Data presented as *n* (%). Groups were compared using the Pearson’s chi-square test or Fisher exact test ^1^.

**Table 6:** Comparison of complications between phaco-iStent and phacocanaloplasty groups.

Variable	Phaco-iStent	Phacocanaloplasty	*p*
Intraoperative and early postoperative complications			
Rupture of trabeculo-descemetic membrane	N/A	2 (2.9)	-
Schlemm’s canal cannulation not possible	N/A	3 (4.3)	-
Microhyphema	2 (2.9)	29 (42.0)	<0.001
Elevated IOP	15 (21.7)	34 (49.3)	0.015 ^1^
Iritis	0 (0.0)	3 (4.3)	0.274
Displacement of iStent	1 (1.4)	N/A	-
Late postoperative complications			
Descemet’s membrane detachment	0 (0.0)	3 (4.3)	0.274
Elevated IOP	10 (14.5)	11 (15.9)	>0.999
Macular edema	2 (2.9)	0 (0.0)	>0.999
Blurry vision or visual disturbance	3 (4.3)	3 (4.3)	>0.999

Notes: IOP, intraocular pressure; N/A, not applicable. Data are presented as *n* (%). Groups were compared using Pearson chi-square ^1^ test or Fisher’s exact test.

## Data Availability

All materials and information will be available upon e-mail request from the corresponding author.
